# Mapping EORTC QLQ-C30 and QLQ-MY20 to EQ-5D in patients with multiple myeloma

**DOI:** 10.1186/1477-7525-12-35

**Published:** 2014-03-11

**Authors:** Irina Proskorovsky, Philip Lewis, Cathy D Williams, Karin Jordan, Charalampia Kyriakou, Jack Ishak, Faith E Davies

**Affiliations:** 1 Evidera, 7575 Trans-Canada Highway, Suite 500, H4T 1V6 Montreal, QC, Canada; 2 Celgene GmbH, Munich, Germany; 3 Centre for Clinical Haematology, Nottingham University Hospital, Nottingham, UK; 4 Klinik und Poliklinik für Innere Medizin IV, Universitätsklinikum Halle (Saale), Halle, Germany; 5 Department of Haematology, Northwick Park and Royal Free Hospitals, London, UK; 6 Institute of Cancer Research and Royal Marsden Hospital, Surrey, UK

**Keywords:** EORTC QLQ-C30, EORTC QLQ-MY20, EQ-5D, Health-related quality of life, Multiple myeloma, Oncology, Utility values

## Abstract

**Background:**

In oncology, health-related quality of life (HRQoL) data are often collected using disease-specific patient questionnaires while generic, patient-level utility data required for health economic modeling are often not collected.

**Methods:**

We developed a mapping algorithm for multiple myeloma that relates HRQoL scores from the European Organization for Research and Treatment of Cancer (EORTC) questionnaires QLQ-C30 and QLQ-MY20 to a utility value from the European QoL-5 Dimensions (EQ-5D) questionnaire. Data were obtained from 154 multiple myeloma patients who had participated in a multicenter cohort study in the UK or Germany. All three questionnaires were administered at a single time point. Scores from all 19 domains of the QLQ-C30 and QLQ-MY20 instruments were univariately tested against EQ-5D values and retained in a multivariate regression model if statistically significant. A 10-fold cross-validation model selection method was also used as an alternative testing means. Two models were developed: one based on QLQ-C30 plus QLQ-MY20 scores and one based on QLQ-C30 scores alone. Adjusted R-squared, correlation coefficients, and plots of observed versus predicted EQ-5D values were presented for both models.

**Results:**

Mapping revealed that Global Health Status/QoL, Physical Functioning, Pain, and Insomnia were significant predictors of EQ-5D utility values. Similar results were observed when QLQ-MY20 scores were excluded from the model, except that Emotional Functioning and became a significant predictor and Insomnia was no longer a significant predictor. Adjusted R-squared values were of similar magnitude with or without inclusion of QLQ-MY20 scores (0.70 and 0.69, respectively), suggesting that the EORTC QLQ-MY20 adds little in terms of predicting utility values in multiple myeloma.

**Conclusions:**

This algorithm successfully mapped EORTC HRQoL data onto EQ-5D utility in patients with multiple myeloma. Current mapping will aid in the analysis of cost-effectiveness of novel therapies for this indication.

## Background

Economic evaluations are an increasingly important aspect of drug development. The cost-utility analysis of a novel therapy is typically assessed in terms of cost per quality-adjusted life-year (QALY) gained, where QALYs reflect both survival and health-related quality of life (HRQoL) [1,2]. Health-state utility values, which represent an individual’s preference for different health outcomes as captured by patient questionnaires, are commonly used by researchers to calculate QALYs. These values are generated by applying an appropriate utility algorithm to the patients’ responses to the questionnaires, and are depicted as an interval scale in which 1 represents perfect health and 0 reflects a health state equivalent to death [1,2]. The most common type of questionnaire used to calculate health-state utility is the EuroQol-5 Dimensions (EQ-5D), a simple questionnaire that takes only a few minutes to complete, and can be applied to patients with any disease type [3]. By providing health-state utility values, preference-based instruments such as the EQ-5D allow health service providers with a means to compare QALYs across different patient groups and disease types, which can aid in decisions regarding broader healthcare resource allocation [1,2].

The measurement of HRQoL in the oncology setting is usually carried out using cancer-specific instruments rather than generic preference-based measures as they focus on relevant health problems, and tend to capture more clinically meaningful differences [4,5]. The use of core questionnaires, such as the European Organization for Research and Treatment of Cancer Quality of Life Questionnaire Core 30 (EORTC QLQ-C30) [6] is standard practice, with these often supplemented with condition-associated modules. Preference-based measures of health status, however, are rarely used. Two recent trials evaluating novel therapies for patients with multiple myeloma, for example, assessed HRQoL using EORTC QLQ-C30 with or without its myeloma-specific module EORTC QLQ-MY20, yet neither trial collected EQ-5D data [6,7]. From an economic perspective, preference-based instruments are required as they can be readily applied at the population level for economic analysis. In the absence of data from preference-based instruments, researchers may utilize suboptimal, non-specific measures, such as clinical response levels, to derive utility values [8].

Mapping algorithms is an alternative means of relating HRQoL scores from core and disease-specific modules to generic utility values. Several studies have demonstrated the feasibility of mapping EORTC HRQoL data to EQ-5D values in cancer patients [4,9-13], including those with breast cancer [10,12], prostate cancer [13], esophageal cancer [4], and gastric cancer [11]. However data on the ability to map EORTC HRQoL data to EQ-5D in patients with multiple myeloma are limited [14,15]. The goal of the current study was to develop a mapping algorithm that uses HRQoL data from the EORTC QLQ-C30 (with or without QLQ-MY20 data) to estimate EQ-5D utility values in patients with multiple myeloma, to facilitate economic evaluation of novel therapies for multiple myeloma.

## Methods

### Data source

HRQoL data from a bi-national, multicenter, cohort study in patients with multiple myeloma were used for this analysis [16]. In addition to information collected from medical charts, supplementary data were collected at the first treatment visit after study enrolment, both from patient interviews and from 3 self-administered HRQoL questionnaires: EOTRC QLQ-C30, EORTC QLQ-MY20, and EQ-5D. Eligible patients, who were aged ≥ 18 years and had a current diagnosis of multiple myeloma, were categorized into one of four pre-defined subgroups at the time of the study visit: asymptomatic, mildly symptomatic, moderately symptomatic, or severely symptomatic. Patients were ineligible if they had undergone autologous stem cell transplantation (ASCT) within the past 3 months or had received experimental treatment from a clinical trial. The study was approved by the institutional review boards of the participating centres and conducted according to the Declaration of Helsinki International Conference on Harmonization and the guidelines for Good Clinical Practice. Written informed consent was obtained from all patients before enrolment.

### Instruments

#### EORTC QLQ-C30

The EORTC QLQ-C30 is a validated 30-item questionnaire containing both single- and multi-item measures [7,17], Additional file 1. These include five functional scales (Physical, Role, Cognitive, Emotional, and Social Functioning), three symptom scales (Fatigue, Pain, and Nausea/Vomiting), a Global Health Status/QoL scale, and six single items (Constipation, Diarrhea, Insomnia, Dyspnea, Appetite Loss, and Financial Difficulties). Scores for each scale and single-item measure are averaged and transformed linearly to a score ranging from 0–100. A high score for functional scales and for Global Health Status/QoL represent better functioning ability or HRQoL, whereas a high score for symptom scales and single items represents significant symptomatology.

#### EORTC QLQ-MY20

The EORTC QLQ-MY20 is a validated instrument, recommended as a supplement to the QLQ-C30 instrument in patients with multiple myeloma [7,18], Additional file 2. The module comprises 20 questions that address four myeloma-specific HRQoL domains: Disease Symptoms, Side Effects of Treatment, Future Perspective, and Body Image. Three of the four QLQ-MY20 domains are multi-item scales: Disease Symptoms (includes bone aches or pain, back pain, hip pain, arm or shoulder pain, chest pain, and pain increasing with activity); Side Effects of Treatment (includes drowsiness, thirst, feeling ill, dry mouth, hair loss, upset by hair loss, tingling hands or feet, restlessness/agitation, acid indigestion/heartburn, and burning or sore eyes); and Future Perspective (includes worry about death and health in the future, and thinking about illness). The Body Image scale is a single-item scale that addresses physical attractiveness. As with the QLQ-C30, QLQ-MY20 domain scores are averaged and transformed linearly to a score ranging from 0–100. A high score for Disease Symptoms and Side Effects of Treatment represents a high level of symptomatology or problems, whereas a high score for Future Perspective and Body Image represents better outcomes.

#### EQ-5D

The EQ-5D is a self-administered questionnaire (Additional file 3) consisting of five dimensions (Mobility, Self-Care, Pain, Usual Activities, and Anxiety/Depression), and a health status rating scale. Each dimension has three levels of “severity” corresponding to the degree of problems encountered: ‘none’, ‘some’, and ‘extreme’. The instrument provides a simple descriptive profile for each participant. Information relating to EQ-5D health states gathered in the context of multinational trials can be converted into a single summary index using one of the available EQ-5D value sets [3]. For this study, UK value sets were used to calculate health utility for all patients. The health utility score can range from −0.594 to 1.0, with 1.0 representing perfect health. The instrument also allows the subject to evaluate his/her current health state on a visual analogue scale that ranges from 0 (worst imaginable health state) to 100 (best imaginable health state).

### Analysis

Multiple linear regression analysis was used to derive a mapping algorithm from EORTC QLQ-C30 and QLQ-MY20 to EQ-5D values. Each scale/item was tested in univariate models against utility. The first multivariate model was then fitted by including scales/items that were found to have a statistically significant association with utility in univariate analysis (*p* < 0.1). The first multivariate model was then manually trimmed down by sequentially removing non-significant predictors with the highest *p*-value until the final model included only significant predictors (*p* < 0.1). Goodness-of-fit of the full model (including all scales of QLQ-C30 and QLQ-MY20) and the model including significant predictors only, was compared using adjusted R-squared values. The predictive ability of the final model was assessed by root mean square error (RMSE) and by comparing predicted and observed EQ-5D utility values. External validation was not possible given the lack of comparable datasets in multiple myeloma in which all three questionnaires were administered. Therefore, as an alternative, a 10-fold cross-validation model selection method was used to assess predictive ability of the mapping. In 10-fold cross-validation, the data are split into 10 approximately equivalent sized parts. The model is fitted on 9 data parts with the 10^th^ being held out for validation. Specifically, the fitted model of the 9 selected parts is used to compute the predicted residual sum of squares on the 10^th^ omitted part, and this process is repeated for each of the 10 parts. The sum of the 10 predicted residual sums of squares is obtained for each fitted model and is the estimate of the prediction error that is denoted by CVPRESS. Among all possible models with varying numbers and combinations of HRQoL scales, the model with the smallest CVPRESS statistic is then selected. The K-fold cross-validation method is commonly used when the aim of the regression model is prediction [19]. Among all candidate models, the model with the smallest CVPRESS statistic has the best predictive ability. In addition, observed vs. predicted EQ-5D values were examined overall and by symptom severity group. This model-building process was repeated to develop a mapping equation based on the QLQ-C30 instrument alone, for application in studies that did not apply the QLQ-MY20 instrument.

## Results

### Patient characteristics, HRQoL scores, and utility values

The study enrolled 154 patients (89 in the UK and 65 in Germany). Baseline patient characteristics are listed in Table 1. Approximately two-thirds of the patients were male, and the average (± standard deviation [SD]) age at enrolment was 66 ± 10 years. Most patients (83%) were of British or German descent. The average time from diagnosis was 3.7 ± 3.7 years, and most patients (88%) had never undergone autologous stem cell transplant (ASCT).

**Table 1:** Baseline patient and disease characteristics

**Variable**	**Overall (N = 154)**
Male – n (%)	97 (63%)
Age – Mean (SD)	66.4 (10.0)
Nationality – n (%)	
British	73 (47%)
German	56 (36%)
Other	25 (16%)
Symptom severity group	
Asymptomatic	17 (11%)
Mildly symptomatic	48 (31%)
Moderately symptomatic	50 (33%)
Severely symptomatic	39 (25%)
Number of co-morbidities present at the time of the visit – n (%)	
None	49 (32%)
1	44 (29%)
2	24 (16%)
3+	37 (24%)
Duration of MM (yrs) – Mean (SD)	3.7 (3.7)
Previous ASCT	18 (12%)

The distribution of EQ-5D, EORTC QLQ-C30, and EORTC QLQ-MY20 scores are summarized in Table 2. The average utility value was 0.7 ± 0.3 and the interquartile range (IQR) was 0.62 to 1.00. The minimum observed utility value was −0.13 and the maximum was 1.0. The mean QLQ-C30 Global Health Status/QoL score was 60.1 ± 25.5 and the IQR was 41.7 to 83.3. Mean Cognitive and Emotional Functioning scores were near or above 80, and these were higher than the scores for Role (62.9 [IQR 33.3 to 100]), Social (63.9 [IQR 33.3 to 100]), and Physical Functioning (68.7 [IQR 53.3 to 93.3]). Mean Pain and Fatigue scores were 32.3 and 38.6, respectively, and mean Insomnia and Dyspnea scores were 25.1 and 21.9, respectively. Diarrhea and Nausea/Vomiting scales had the lowest mean scores (< 10). For the QLQ-MY20 instrument, Body Image scores were generally high (77.9 [IQR 66.7 to 100]), but Future Perspective scores appeared to be relatively more affected (59.9 [IQR 33.3 to 77.8]). The Disease Symptom (23.3 [IQR 0 to 38.9]) and Side Effect scale scores (19.5 [IQR 7.4 to 29.6]) were roughly consistent in numbers with the symptom scales from the QLQ-C30.

**Table 2:** Distribution of EQ-5D, EORTC QLQ-C30 and EORTC QLQ-MY20 scores

	**No. of patients**	**Mean ± SD**	**Median**	**Interquartile range**
*EQ-5D*	154	0.7 ± 0.3	0.73	0.62–1.00
*EORTC QLQ-C30*				
Global health status/QoL	154	60.1 ± 25.5	58.3	41.7–83.3
Functional scales				
Physical	154	68.7 ± 27.2	73.3	53.3–93.3
Role	153	62.9 ± 34.6	66.7	33.3–100.0
Emotional	154	78.1 ± 24.6	83.3	66.7–100.0
Cognitive	154	81.4 ± 22.9	83.3	66.7–100.0
Social	154	63.9 ± 32.9	66.7	33.3–100.0
Symptom scales				
Fatigue	154	38.6 ± 29.8	33.3	11.1–66.7
Nausea/Vomiting	154	5.2 ± 11.8	0.0	0.0–0.0
Pain	154	32.3 ± 33.4	16.7	0.0–66.7
Single items				
Dyspnea	154	21.9 ± 30.6	0.0	0.0–33.3
Insomnia	154	25.1 ± 29.8	16.7	0.0–33.3
Appetite Loss	154	15.4 ± 27.0	0.0	0.0–33.3
Constipation	154	17.7 ± 28.6	0.0	0.0–33.3
Diarrhea	154	8.4 ± 21.7	0.0	0.0–0.0
Financial difficulties	154	18.4 ± 31.2	0.0	0.0–33.3
*EORTC QLQ-MY20*				
Disease symptoms	154	23.3 ± 22.3	16.7	0.0–38.9
Side-effects of treatment	154	19.5 ± 17.1	14.8	7.4–29.6
Future perspective	154	59.9 ± 28.1	66.7	33.3–77.8
Body image	154	77.9 ± 30.5	100.0	66.7–100.0

*SD* standard deviation.

### Mapping QLQ-C30 and QLQ-MY20 to EQ-5D

**Figure 1:**
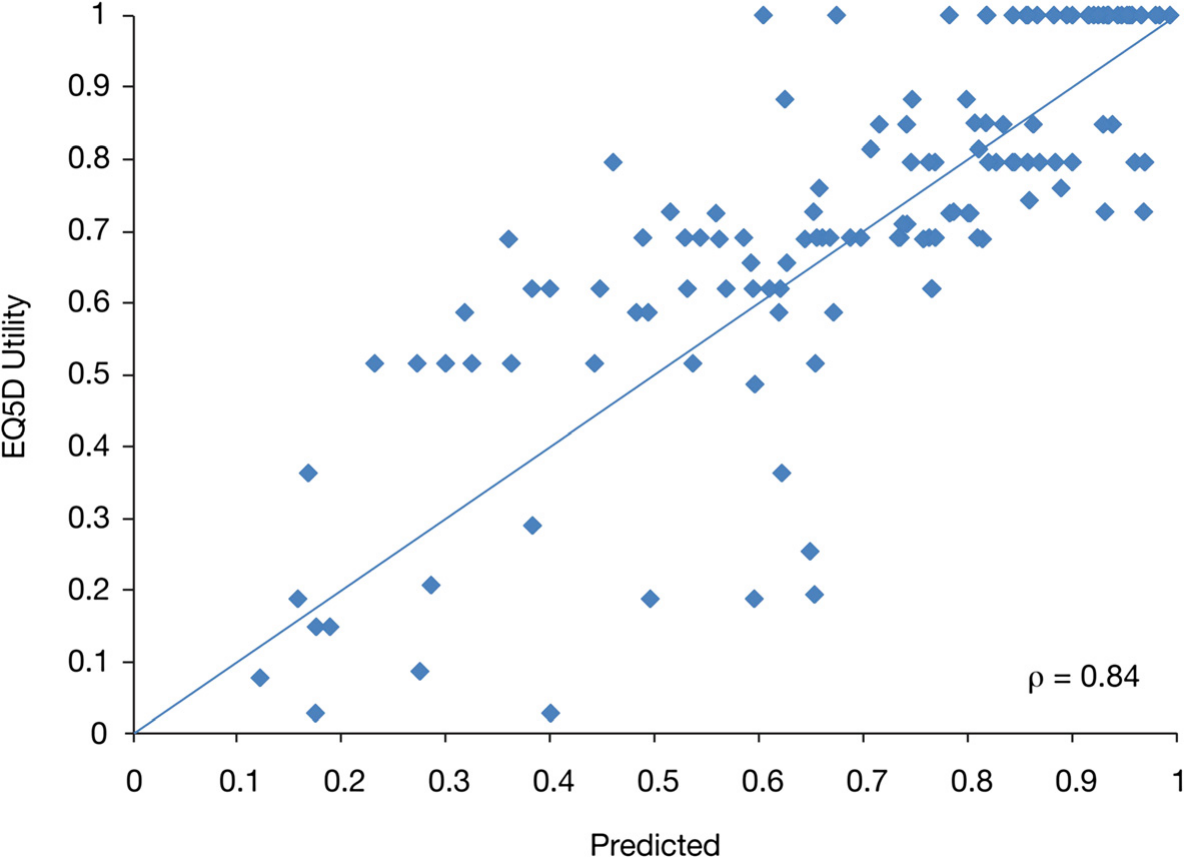
Observed and predicted EQ-5D utility from trimmed model with EORTC QLQ-C30 and QLQ-MY20.

Results from multiple regression analysis are summarized in Table 3. Full model results, which included all scales and individual items from both EORTC QLQ-C30 and EORTC QLQ-MY20, had an adjusted R-squared value of 0.7015. A trimmed model that included only significant predictors had an adjusted R-squared value of 0.7028, suggesting that the trimmed model fits the data as well as the full model, and both models explain a large part of the observed variation in EQ-5D scores. The final trimmed model included Global Health Status/QoL, Physical Functioning, Pain, Insomnia and Future Perspective. Better Global Health Status/QoL and Physical Functioning and Future Perspective were associated with higher utility values, as was Insomnia. Pain had a significant negative association with utility values. The full and trimmed models had very similar predictive ability with RMSE indices of 0.164 and 0.163, respectively. The trimmed model also had the lowest CVPRESS statistic as generated during the validation phase, thus highlighting its best predictive performance amongst all candidate models tested. A plot of observed versus predicted utility scores from the trimmed model indicates that the model fits the data well (Pearsons correlation coefficient = 0.84; Figure 1). Differences between observed and predicted mean EQ-5D utility values by symptom subgroup from the QLQ-C30 + MY20 model are also presented in Table 4. The mean difference was smallest for the moderately symptomatic patients (−0.004) and for all symptom groups the predicted values did not deviate by more than 0.030 from the observed values. Observed utility values for all symptom groups were within 95% CI of the predicted mean utility.

**Table 3:** Multiple regression analyses mapping EORTC QLQ-C30 and EORTC QLQ-MY20 data to EQ-5D

**Predictors**	**Full model**		**Trimmed model**	
	**Estimate**	***p*-value**	**Estimate**	***p*-value**
QLQ-C30				
Intercept	0.11979	0.3757	0.25763	0.0002
Global health status/QoL*	0.00161	0.0568	0.00165	0.0301
Physical functioning*	0.00471	< 0.0001	0.00467	< 0.0001
Role functioning	0.00073194	0.3455	NS	NS
Emotional functioning	0.00104	0.2522	NS	NS
Cognitive functioning	−0.00037864	0.6203	NS	NS
Social functioning	0.00044712	0.4983	NS	NS
Fatigue	−0.00005750	0.9521	NS	NS
Nausea/Vomiting	0.00053330	0.7166	NS	NS
Pain*	−0.00229	0.0039	−0.00293	< 0.0001
Dyspnoea	0.00037610	0.5099	NS	NS
Insomnia*	0.00096618	0.0570	0.00089197	0.0616
Appetite loss	−0.00047987	0.4899	NS	NS
Constipation	−0.00055193	0.3057	NS	NS
Financial difficulties	0.00096199	0.0613	NS	NS
QLQ-MY20				
Disease symptoms	−0.00094431	0.3983	NS	NS
Side effects	0.00207	0.1445	NS	NS
Future perspective*	0.00149	0.0366	0.00157	0.0061
Body image	−0.00040870	0.4607	NS	NS
Adjusted R-squared values	0.7015		0.7028	
RMSE indices	0.164		0.163	

*Significant predictors in the full model.

*NS* not significant.

**Table 4:** Observed vs. Predicted EQ-5D Utility by symptom group from QLQ-C30 + MY20 model

	**Actual**	**Predicted (95% CI)**	**Difference**
Symptom group			
Asymptomatic	0.923	0.893 (0.819; 0.967)	0.030
Mildly symptomatic	0.806	0.833 (0.783; 0.883)	−0.027
Moderately symptomatic	0.675	0.679 (0.616; 0.743)	−0.004
Severely symptomatic	0.501	0.474 (0.398; 0.551)	0.027

### Mapping QLQ-C30 to EQ-5D

**Figure 2:**
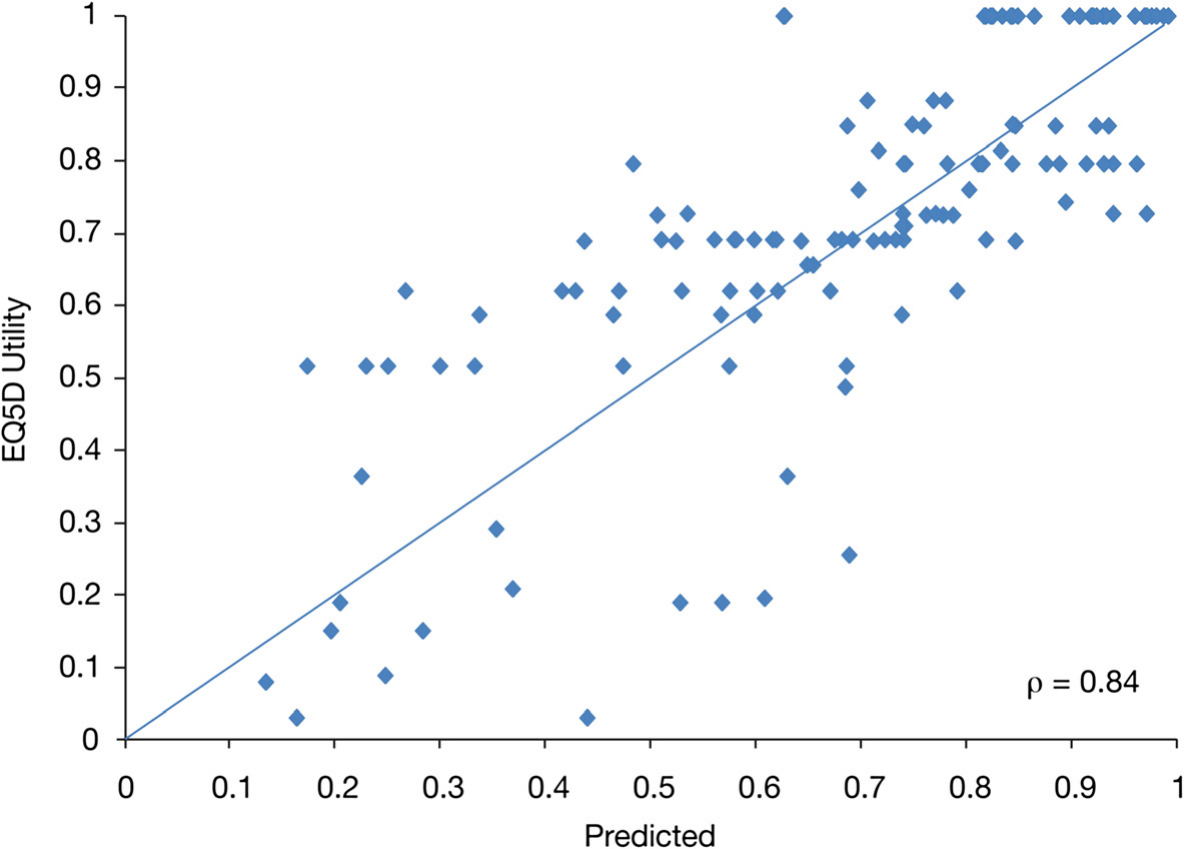
Observed and predicted EQ-5D utility from trimmed model with EORTC QLQ-C30 only.

Results from multiple regression analysis using EORTC QLQ-C30 data only, are summarized in Table 5. The final trimmed model included Global Health Status/QoL, Physical Functioning, Emotional Functioning, and Pain (Table 5). Better Global Health Status/QoL, Physical Functioning, and Emotional Functioning were associated with higher utility values, whereas a higher Pain score (i.e., worse pain) was associated with lower utility values. Both models had similar and good explanatory power (adjusted R-squared values of 0.6956 for the full model and 0.6941 for the trimmed model). Predictive ability of both models was also comparable (RMSE of 0.165 for both the full and trimmed models). Again, the trimmed model had the lowest CVPRESS, suggesting it has optimal generalizability compared with all other iterations tested during the validation phase. A plot of observed versus predicted utility scores from the trimmed model indicates that the model fits the data well (Pearsons correlation coefficient = 0.84; Figure 2). Differences between observed and predicted mean EQ-5D utility values by symptom subgroup from the QLQ-C30 model only, are presented in Table 6. The mean difference was smallest for the moderately symptomatic subgroup of patients (−0.007) and, for all subgroups, predicted values did not deviate by more than 0.031 from observed values. Observed utility values for all symptom severity groups were all within 95% CI of predicted mean utility.

**Table 5:** Multiple regression analyses mapping QLQ-C30 data to EQ-5D

**Predictors**	**Full model**		**Trimmed model**	
	**Estimate**	***p*-value**	**Estimate**	***p*-value**
Intercept	0.15540	0.2192	0.23004	0.0042
Global health status/QoL*	0.00198	0.0180	0.00191	0.0106
Physical functioning*	0.00463	< 0.0001	0.00478	< 0.0001
Role functioning	0.00058079	0.4512	NS	NS
Emotional functioning*	0.00141	0.0696	0.00136	0.0405
Cognitive functioning	−0.00048664	0.5075	NS	NS
Social functioning	0.00059878	0.3536	NS	NS
Fatigue	0.00016137	0.8588	NS	NS
Nausea/Vomiting	0.00041262	0.7764	NS	NS
Pain*	−0.00249	0.0001	−0.00249	< 0.0001
Dyspnea	0.00060165	0.2879	NS	NS
Insomnia	0.00082466	0.1039	NS	NS
Appetite loss	−0.00037029	0.5885	NS	NS
Constipation	−0.00050445	0.3468	NS	NS
Financial difficulties	0.00079559	0.1187	NS	NS
Adjusted R-squared values	0.6956		0.6941	
RMSE indices	0.165		0.165	

*Significant predictors in the full model.

*NS* not significant.

**Table 6:** Observed vs. Predicted EQ-5D Utility by Symptom Group from QLQ-C30 Model

	**Actual**	**Predicted (95% CI)**	**Difference**
Symptom group			
Asymptomatic	0.923	0.892 (0.816; 0.968)	0.031
Mildly symptomatic	0.806	0.830 (0.780; 0.880)	−0.024
Moderately symptomatic	0.675	0.682 (0.617; 0.747)	−0.007
Severely symptomatic	0.501	0.476 (0.404; 0.548)	0.025

## Discussion

This study reports the mapping of EORTC QLQ-C30 scores (with and without QLQ-MY20 scores) to EQ-5D utility values in patients with multiple myeloma. The ability to estimate utility values based on HRQoL data is beneficial in this setting since recent major trials of novel agents in multiple myeloma have not collected preference-based data [6,7]. Although the information on physical and mental health provided by EORTC QLQ-C30 and QLQ-MY20 questionnaires offer great insight to clinicians, the questionnaires are not pertinent to cost-utility analyses as they are not easily translated into global health-utility values.

Mapping algorithms is an alternative means of relating HRQoL scores from core and disease-specific modules to generic utility values, and similar mappings have been developed in other cancer populations. In a study of 48 patients with gastric cancer, Kontodimopoulos and colleagues [11] demonstrated the ability of QLQ-C30 scores to predict 15D, Short Form-6D (SF-6D) and, to a lesser extent, EQ-5D utility values. McKenzie and Van der Pol [4] mapped QLQ-C30 scores to EQ-5D in 199 patients with inoperable esophageal cancer in a similar type of study using regression analysis techniques. In both studies, however, the model fit was somewhat lower (adjusted R-square 0.61 for both) compared with current mapping (adjusted R-square of 0.69). In a study of 280 patients with hormone-refractory prostate cancer, Wu and colleagues [13] developed models to predict EQ-5D utility values based on QLQ-C30, Functional Assessment of Cancer Therapy-Prostate (FACT-P) scores, and patient demographics using ordinary least square regression analysis. However, this mapping can only be used when both QLQ-C30 and FACT-P scores are collected within the same study.

Two previous studies have reported mapping algorithms involving multiple myeloma. Rowen and colleagues [14] derived utility values from QLQ-C30 scores in patients with multiple myeloma, but used a custom-designed preference-based measure (EORTC-8D), rather than the more generic EQ-5D. Versteegh and colleagues [15] mapped QLQ-C30 scores to EQ-5D using a dataset derived from a HOVON trial of patients with previously untreated multiple myeloma (HOVON 24); the predictive value of this model was validated in a population of patients with non-Hodgkin lymphoma (HOVON 25). In this model, including only significant predictors, all other domains of QLQ-C30 were the same as in our study, however the model fit was not as good (R-square 0.51). Neither of the two mapping studies in multiple myeloma addressed the contribution of EORTC QLQ-MY20 scores.

The HRQoL scales identified in this analysis as significant predictors of utility values, such as Global Health Status/QoL, Physical Functioning, and Pain, are similar to HRQoL scales that have been pre-selected as clinically relevant in previous assessments [7,20]. Our final model included Global Health Status/QoL, Physical Functioning, Pain, Insomnia, and Future Perspective when both QLQ-C30 and QLQ-MY20 were used. Using QLQ-C30 alone, the final model also included Emotional Functioning alongside Global Health Status/QoL, Physical Functioning, and Pain. The adjusted R-squared value for both models was around 0.70, signifying a strong association and predictability. The high R-squared values and correlation between observed and predicted utility values of 0.84, suggest that the algorithm performs very well. A limitation to the above findings is the reverse association found between Insomnia and EQ-5D (i.e. worse insomnia associated with higher utility) when testing HRQoL domains from both the QLQ-C30 and QLQ-MY20 instruments. Unlike all other statistical associations, this is a counterintuitive finding. Insomnia was no longer a significant predictor when only QLQ-C30 domains were used as predictors for EQ-5D. Beside the almost equal predictability of HRQoL domains from the QLQ-C30 alone as compared to testing of HRQoL domains from both the QLQ-C30 and the QLQ-MY20, this provides an additional argument for using the QLQ-C30 as a stand-alone instrument for mapping onto EQ-5D. Table 2 also indicates possible floor effects on disease-specific symptom scores Dyspnea, Constipation, Appetite Loss, Diarrhea, and Nausea/Vomiting, all with median scores of 0.0, suggesting that these symptoms were not perceived at all by more than half of all patients. The five above-mentioned disease-specific symptoms are known to be reported in the context of novel treatments, and it is therefore possible that these scores in particular have been underreported in the context of this study. However, since our model displays comparably strong predictive power, independent of the symptom group chosen (see Tables 4 and 6), this does not reduce the predictability of the mapping algorithm presented here. Finally, further validation of the predictive performance of the algorithms presented here using external containing all three instruments is recommended.

## Conclusion

The derived mapping algorithm establishes a link between EORTC HRQoL data and the EQ-5D as a utility-based outcomes measure specifically in patients with multiple myeloma. The results of the study are very encouraging in terms of the predicting power of mappings from QLQ-C30 scores alone or in combination with QLQ-MY20. This mapping algorithm will be a beneficial tool for deriving utility values from data obtained using the QLQ-C30 with or without the myeloma-specific instrument, and will thus enable to conduct cost-utility analyses. From a clinical perspective, the results may provide good guidance with regards to HRQoL domain selection for the purpose of primary analysis. Given the quantity of HRQoL data typically generated, a detailed discussion on all available HRQoL domains is often difficult to accomplish in the context of peer-reviewed publications. The results presented here provide an indication of the HRQoL domains that may be of particular interest in best describing a myeloma patient’s general health state and quality of life.

## Competing interests

IP and JI are employed by Evidera which provides consulting and other research services to pharmaceutical, device, government, and non-government organizations. In this position, they work with a variety of companies and organizations; no payment or honoraria directly from these organizations for services rendered is received. PL is employed by and holds stock options in Celgene. The remaining authors have declared no conflicts of interest relevant to this study.

## Authors’ contributions

IP, PL and CDW conceived and designed the study. CDW, KJ and FED collected and assembled data. PL, JI, CK, CDW, KJ and FED analyzed and interpreted data. CK, CDW and KJ assisted with the provision of materials and patients. All authors wrote and approved the manuscript.

## Supplementary Material

Additional file 1 **EORTC QLQ-C30**^**© **^**questionnaire (Aaronson NK, Ahmedzai S, Bergman B, et al. for the European Organization for Research and Treatment of Cancer QLQ-C30: A Quality-of-Life Instrument for Use in International Clinical Trials in Oncology.** JNCI 85: 365–376, 1993) [17]. Additional files 1 and 2 are copyrighted and the Intellectual Property rights belong to EORTC. EORTC’s material does not infringe upon the copyright or other rights of anyone. For use please contact the Quality of Life Department directly (http://groups.eortc.be/qol/). Click here for file

Additional file 2 **EORTC QLQ-MY20**^**© **^**questionnaire **[18]**.** Click here for file

Additional file 3 **EQ-5D questionnaire **[3]^**©**^**.** UK (English) © 1990 EuroQol Group EQ-5D™ is a trade mark of the EuroQol Group. For use please contact the EuroQol Group (http://www.euroqol.org). Click here for file
