# Supplementary material for: Mapping EORTC QLQ-C30 and QLQ-MY20 to EQ-5D in patients with multiple myeloma
Source: Health Qual Life Outcomes. 2014 Mar 11;12:35. doi: 10.1186/1477-7525-12-35 (PMC4007827; doi:10.1186/1477-7525-12-35)
Supplement: Additional file 2 — EORTC QLQ-MY20 © questionnaire [18]. [file 1477-7525-12-35-S2.pdf]

EORTC QLQ – MY20

Patients sometimes report that they have the following symptoms or problems. Please indicate the extent to which you have experienced these symptoms or problems during the past week. Please answer by circling the number that best applies to you.

During the past week:	Not at All	A Little	Quite a Bit	Very Much
31. Have you had bone aches or pain?	1	2	3	4
32. Have you had pain in your back?	1	2	3	4
33. Have you had pain in your hip?	1	2	3	4
34. Have you had pain in your arm or shoulder?	1	2	3	4
35. Have you had pain in your chest?	1	2	3	4
36. If you had pain did it increase with activity?	1	2	3	4
37. Did you feel drowsy?	1	2	3	4
38. Did you feel thirsty?	1	2	3	4
39. Have you felt ill?	1	2	3	4
40. Have you had a dry mouth?	1	2	3	4
41. Have you lost any hair?	1	2	3	4
42. Answer this question only if you lost any hair: Were you upset by the loss of your hair?	1	2	3	4
43. Did you have tingling hands or feet?	1	2	3	4
44. Did you feel restless or agitated?	1	2	3	4
45. Have you had acid indigestion or heartburn?	1	2	3	4
46. Have you had burning or sore eyes?	1	2	3	4

Please turn to next page

SPECIMEN

During the past week:	Not at All	A Little	Quite a Bit	Very Much
47. Have you felt physically less attractive as a result of your disease or treatment?	1	2	3	4
48. Have you been thinking about your illness?	1	2	3	4
49. Have you been worried about dying?	1	2	3	4
50. Have you worried about your health in the future?	1	2	3	4

SPECIMEN

© Copyright 1999 EORTC Study Group on Quality of Life. All rights reserved.
